# Supplementary material for: Testosterone and Obesity in an Aging Society
Source: Biomolecules. 2025 Oct 28;15(11):1521. doi: 10.3390/biom15111521 (PMC12650755; doi:10.3390/biom15111521)
Supplement: Supplementary file 1 [file biomolecules-15-01521-s001.zip › biomolecules-3904906-supplementary.pdf]

Figure S1.

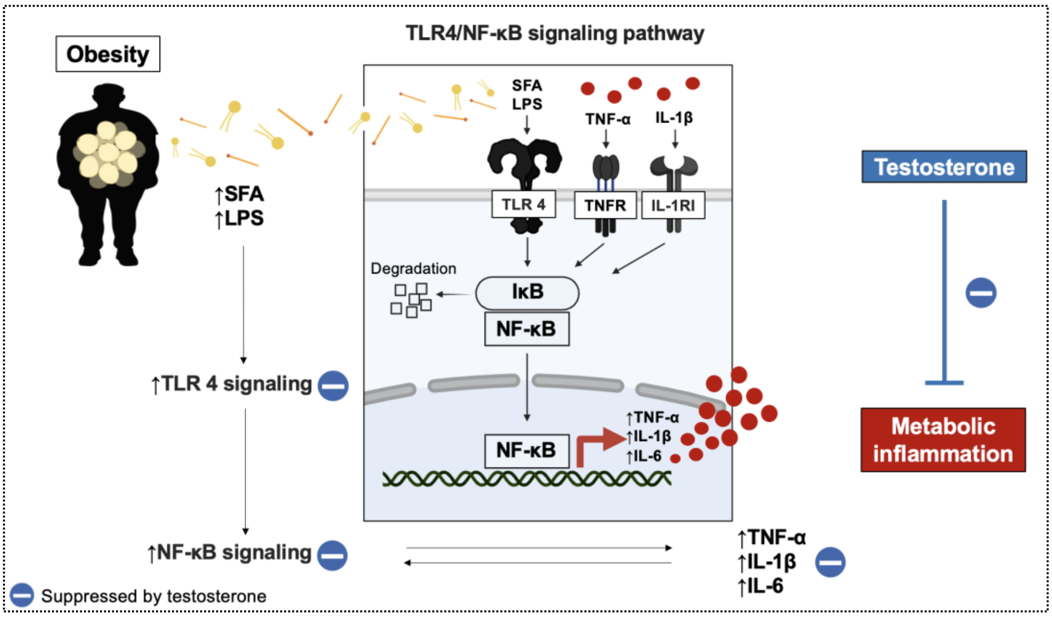

Suppression of Metabolic Inflammation by Testosterone

Obesity increases the production of Toll-like receptor 4 (TLR4) ligands such as saturated fatty acids (SFA) and lipopolysaccharide (LPS), thereby activating TLR4 signaling. This activation leads to the degradation of IκB, resulting in subsequent activation of NF-κB signaling. Consequently, the production of inflammatory cytokines including TNF-α, IL-1β, and IL-6 is elevated, ultimately inducing systemic metabolic inflammation. Furthermore, TNF-α and IL-1β enhance NF-κB signaling through their respective receptors, TNF receptor (TNFR) and interleukin-1 receptor type I (IL-1RI). Testosterone may suppress TLR4 and NF-κB signaling, as well as the production of inflammatory cytokines, thereby potentially attenuating metabolic inflammation.

Abbreviations: Saturated fatty acids (SFA); Lipopolysaccharide (LPS); Inhibitor of κB (IκB); TNF receptor (TNFR); Interleukin-1 receptor type I (IL-1RI).
